# Supplementary material for: Comparison of the performance of four clinical prediction rules for mortality in patients with COVID-19
Source: PLoS One. 2026 May 14;21(5):e0348683. doi: 10.1371/journal.pone.0348683 (PMC13175323; doi:10.1371/journal.pone.0348683)
Supplement: S1 Table — This table details the availability of each variable required for score calculation and identifies the primary laboratory or clinical parameters limiting the analysis for each model. (DOC) [file pone.0348683.s001.doc]

**Table S1. Feasibility and data completeness across the four clinical prognostic scores (N** = 1,963).

| **Score** | **Complete cases (n)** | **Missing data** | **Completeness (%)** | **Primary variable limiting calculation (n missing)** |
| --- | --- | --- | --- | --- |
| **qCSI** | 1,844 | 119 | 93.9% | Respiratory Rate (n=119) |
| **ISARIC-4C** | 1,408 | 555 | 71.7% | C-Reactive Protein (n=319) |
| **SEIMC** | 1,792 | 171 | 91.3% | eGFR (n=171) |
| **CALL** | 1,420 | 543 | 72.3% | LDH (n=543) |

N, total study population; n, number of patients with available data. Completeness (%) was calculated as (n/N) x 100. Primary variables responsible for score exclusion included: Respiratory Rate (for qCSI); C-Reactive Protein (for ISARIC-4C); estimated Glomerular Filtration Rate (eGFR, for SEIMC); and Lactate Dehydrogenase (LDH, for CALL). Cases with missing values for any component variable were excluded from the final score calculation.
