# Supplementary material for: Comparison of the performance of four clinical prediction rules for mortality in patients with COVID-19
Source: PLoS One. 2026 May 14;21(5):e0348683. doi: 10.1371/journal.pone.0348683 (PMC13175323; doi:10.1371/journal.pone.0348683)
Supplement: S2 Table — This table compares the final analytic cohort (n = 1,074) with patients excluded due to missing data (n = 889) to assess potential selection bias across demographic and clinical variables. (DOC) [file pone.0348683.s002.doc]

**Table S2. Comparison of baseline characteristics between included (Complete scores) and excluded p**atients.

| **Characteristics** | **Included (n=1074)** | **Excluded (n=889)** | **P-value** |
| --- | --- | --- | --- |
| **Age (years), mean (SD)** | 58.2 ± 14.81 | 56.4 ± 16.03 | 0.011^a^ |
| **Sex, n(%)**  Male  Female | 729 (67.9 %)  345 (32.1 %) | 574 (64.6 %)  315 (35.4 %) | 0.122^b^ |
| **Oxygen Saturation (%), median (IQR)** | 88 (80-93) | 88 (80-93) | 0.182^c^ |
| **Comorbidities, n (%)**  Diabetes  Hypertension | 300 (27.9 %)  189 (17.6 %) | 202 (22.7 %)  176 (19.8 %) | 0.008^b^  0.212^b^ |

SD: Standard Deviation; IQR: Interquartile Range. ^a^ Welch's t-test; ^b^ Pearson's Chi-square test; ^c^ Mann-Whitney U test.
