# Supplementary material for: Comparison of the performance of four clinical prediction rules for mortality in patients with COVID-19
Source: PLoS One. 2026 May 14;21(5):e0348683. doi: 10.1371/journal.pone.0348683 (PMC13175323; doi:10.1371/journal.pone.0348683)
Supplement: Attachment — Submitted filename: Response to Reviewers.doc [file pone.0348683.s006.doc]

**A rebuttal letter**

Dear Frederick K Wangai, Academic Editor, and anonymous reviewers,

Thank you sincerely for your time, the thorough reading of our manuscript (PONE-D-25-29570), and the constructive and insightful comments you have provided. We believe that your suggestions and critiques have significantly strengthened the clarity and scientific rigor of our work.

We have carefully reviewed every point raised by the Academic Editor and the Reviewers, and we have made all necessary modifications in the revised manuscript.

In the following "Response to Reviewers Letter," we address each observation point-by-point. To facilitate the review process, we have copied and pasted the original comments in bold and italics and have presented our response immediately afterward, indicating the changes made in the manuscript (including the page or line number where applicable).

We hope that the revisions fulfill the high standards of *PLOS ONE,* and we thank you in advance for your consideration regarding the publication of our work.

Sincerely,

**Reviewer #1**

***Reviewer's observation #1. This is an excellent manuscript and thou the statistics at first glance appear complicated it simply and clearly explained. Well done.
Just to be sure,***

***Response:*** *We are highly gratified that the statistical methodology, while potentially appearing complex at first glance, was perceived as “simply and clearly explained.” We sincerely thank the reviewer for this positive assessment.*

***Reviewer's observation #2. Line 84: should it read referral or reference ?***

**Response:** We appreciate this important point regarding terminology. We have reviewed Line 84 of the original manuscript and confirm that, in the context of patient transfer or moving a patient between different levels of care, the correct term is “referral”. We have corrected the wording in the manuscript to ensure precision and clarity.

**Reviewer #2**

***1. Selection Bias and Missing Data Handling: Only 1,074 of 3,074 patients (≈35%) had complete data for calculation of all four scores. Excluding two-thirds of patients risks selection bias. Missing data may not be random, and patients with incomplete records could differ systematically (e.g., severity, comorbidities, outcomes). The authors should compare baseline characteristics of included versus excluded patients and discuss how these differences might bias estimates. It would also be useful to explain why the sample sizes for each score (n=1844 for q‑CSI, 1408 for ISARIC‑4C, etc.) are larger than the final sample of 1,074-likely because each score had different missing variables. Clarifying whether multiple imputations or other strategies were considered would strengthen validity.***

**Response:**

We sincerely appreciate your detailed and highly pertinent observation regarding the potential selection bias arising from the high proportion of missing data in our retrospective cohort.

1. We fully concur that the exclusion of a significant portion of the original patient cohort represents a limitation and introduces risk of selection bias. This high rate of data incompleteness is a direct reflection of the extreme resource constraints and clinical burden faced by our hospital, and the Peruvian health system overall, during the first wave of the pandemic. The resulting compromise in the quality of clinical documentation retrospectively underscores the pressing need for improved data capture systems in low-resource settings -a limitation that we feel merits explicit discussion in the manuscript, as it represents a negative impact on the quality of healthcare records.

2. Although 3074 patients were hospitalized, 2377 satisfied our general inclusion criteria. Among them, 1074 had compete information for calculating all four scores under evaluation (see flowchart). While we recognize the value of your suggestion to compare the baseline characteristics (demographics, comorbidities, and mortality outcome) between the patient with (n=1,074) and without complete information (n=1,303), we must regretfully report that this analysis is not methodologically feasible. The exclusion process occurred early during the data abstraction phase: patients whose charts were missing the minimum variables required for any of the four prediction rules were immediately filtered out. Consequently, their full baseline clinical characteristics and, critically, their mortality outcomes, were not extracted or digitized, precluding any comparison.

3. As the reviewer pointed out, the sample sizes for each individual score are larger than the final sample of 1,074 because each score had different missing variables.

To provide clarity on the distinct sample sizes (n) reported:

- n=1,074 (Primary cohort): This is the core comparative cohort. It is defined strictly by patients who had complete data necessary to calculate ALL four prediction scores concurrently (q-CSI, ISARIC-4C, SEIMC, and CALL). This group ensures a robust head-to-head comparison of all models.
- Larger “n” for Individual Scores: The larger sample sizes cited for individual scores (e.g., n=1,844 for q-CSI) reflect the differential availability of the specific variables required for each model. The q-CSI, requiring fewer and more common clinical and basic laboratory parameters, was applicable to a significantly larger subset of the initial pool compared to the ISARIC-4C, which relies on more complex inflammatory markers often missing from retrospective charts. This demonstrates that simpler scores are inherently more robust to data incompleteness in our setting.

4. Regarding alternative strategies, we also considered methods such as multiple imputation. However, given that the percentage of excluded patients (1,303 out of 2,377) represents 55% of the eligible cohort, we determined that multiple imputation would not yield statistically robust estimates. We therefore opted for the complete-case analysis, explicitly acknowledging and discussing its inherent limitations in the Discussion section.

Actions in the Manuscript:

- We will integrate these essential clarifications into the revised methods and discussion sections of the manuscript.

***2. The cohort is single-center and exclusively unvaccinated. Given vaccination and new variants significantly change disease presentation and outcomes, the findings may not apply to contemporary COVID‑19 patients. The authors acknowledge this limitation but should expand discussion of how vaccination status, variant virulence, and changing treatment protocols affect predictive scores. Additionally, Peru’s healthcare system and patient demographics may differ from other Latin American and global contexts, limiting extrapolation.***

**Response:**

We thank the reviewer for raising this concern. We acknowledge that the generalizability of our findings is a key limitation due to the study's specific temporal and geographical context.

1. We accept that the single-center nature of our cohort, coupled with the fact that the study was conducted exclusively in unvaccinated patients during the initial waves of the pandemic (March to December 2020), inherently restricts the direct applicability of our results to current clinical practice. It is well-established that the development of population immunity, the emergence of new viral variants, and the standardization of therapeutics (such as corticosteroids and antivirals) have substantially altered the natural history, clinical presentation, and mortality risk of COVID-19. This evolution may indeed affect the predictive performance of scores developed or validated in earlier phases. We have expanded upon this critical point in the Discussion section.

2. Nevertheless, we believe the value of this study remains significant for several reasons:

- This validation study focuses on a Latin American (Peruvian) cohort, a region historically underrepresented in prognostic score validation literature. This provides essential data on the performance of these tools within a specific demographic and socioeconomic context.
- Our finding that simpler scores (like q-CSI) maintained high performance is crucial for triage and decision-making in low-resource environments, where access to complex laboratory variables necessary for scores like ISARIC-4C is often a significant logistical bottleneck.
- Data from unvaccinated or immunologically naive populations hold paramount importance for planning and response strategies against future public health emergencies that may involve novel pathogens or populations lacking prior immunity.

3. Finally, we will re-emphasize that the clinical application of these scores must always be understood within the context of hospitalized patient risk stratification. By design, their extrapolation to the general population (ambulatory patients or community triage) is, and remains, limited.

Actions in the Manuscript:

- The Discussion section will be expanded to incorporate a more detailed analysis of the impact of vaccination, variants, and evolving treatments on the predictive capacity of the scores.
- The justification for the study will be reinforced, highlighting the geographical gap and the utility for resource-limited settings.

***3. Retrospective Data Quality: Retrospective chart reviews risk misclassification and documentation errors. The authors mention manual review of physical charts and data transfer to Excel, but further details about data quality control, inter-rater reliability, and training of abstractors would enhance confidence in the dataset.***

**Response:**

We agree that data quality is an essential component of methodological rigor and that retrospective review, particularly in a public health emergency context, carries an inherent risk of transcription errors or misclassification.

To ensure the quality and reliability of our dataset, we implemented a comprehensive control protocol:

1. Data abstraction was carried out by the same core team of investigators who were simultaneously working as treating physicians in the hospitalization areas designated for COVID-19 patients. Their extensive clinical experience and direct knowledge of the clinical context were crucial for the correct and consistent interpretation of clinical and laboratory variables (including those obtained from handwritten records).

2. For rigorous transcription quality control, 100% of the abstracted data was subjected to a double data entry process (double digitization). Two team members independently entered the information into separate electronic files. Subsequently, a systematic cross-verification was performed to detect all discrepancies. Any differences identified between the two entries were immediately reviewed by a senior investigator and resolved by referring to the original patient record. This process resulted in a single, final validated dataset.

3. While we acknowledge that a formal measurement of inter-rater reliability was not performed, we consider this limitation to be substantially mitigated. Our confidence in the data quality is based on the unique combination of the deep clinical experience and contextual knowledge held by the physician/investigator abstractors, coupled with the methodological rigor of the systematic double data entry and cross-verification process.

Actions in the Manuscript:

- These specific details concerning the composition of the investigation team (treating physicians/investigators), double data entry, and the cross-verification protocol will be incorporated into the "Methods: Data Collection" section to enhance methodological transparency.

**4. Calibration and Model Updating: Although discrimination is the focus, calibration is crucial for clinical use. The q‑CSI, SEIMC, and CALL scores demonstrated poor Hosmer–Lemeshow fit, indicating risk predictions deviate from observed probabilities. The authors might explore recalibration or model updating tailored to their cohort. For example, logistic recalibration or refitting intercepts and slopes could improve predictive accuracy (Van Calster et al. 2019). If recalibration is out of scope, at least provide calibration intercepts and slopes or net reclassification improvement measures; these metrics are more informative than Hosmer–Lemeshow, which is sensitive to sample size (Riley et al. 2019).**

**Response:**

We thank the Reviewer for highlighting the importance of calibration and for guiding us towards the use of more informative metrics. We agree that the Hosmer-Lemeshow (HL) test is highly sensitive to large sample sizes, potentially masking adequate calibration performance.

We have addressed this point comprehensively by calculating and reporting the Calibration Intercept (alpha) and the Calibration Slope (beta) for all four scores in the complete case cohort (n=1,074).

1. Our analysis using alpha and beta demonstrates that the global calibration of all four models was robust, in contrast with the conclusion of 'poor fit' derived solely from the HL test.

- Calibration Slope (beta): The slope for all four scores was found to be statistically indistinguishable from the ideal value of 1.0. This demonstrates that the risk scale is structurally correct in our cohort.
- Calibration Intercept (alpha): The alpha values for the SEIMC (alpha=-0.04) and CALL (alpha=-0.03) scores were virtually zero. While the q-CSI (alpha=-0.14) and ISARIC (alpha=-0.13) showed a slight negative trend (suggesting minor risk overestimation), this deviation was not statistically significant (p > 0.05 in all cases).

We have expanded the Discussion to emphasize that these robust metrics (Table 2) confirm the models are globally well-calibrated, validating their use for risk stratification despite the negative HL result.

2. We acknowledge the importance of recalibration (e.g., Logistic Recalibration, as suggested by Van Calster et al.) for maximizing local utility.

- Since our primary objective was the direct external validation and comparison of the published models’ original performance, model updating or recalibration falls outside the scope of this initial study. However, we have made this explicit recommendation for future research based on the minor local deviations observed in the calibration plots.

3. Regarding the suggested to include the references by Van Calster et al. 2019 and Riley et al. 2019.

We believe the Van Calster et al. reference focuses on the methodology of recalibration and model updating, which falls outside the scope of our validation study. We regret to inform that despite efforts to locate the full text, we were unable to gain access to the work by Riley et al. (2019) to integrate its methodology. Therefore, we will instead cite the principles of calibration assessment based on available methodological literature (Stevens RJ et al 2020).

Actions in the Manuscript:

- The global performance table (table 2) in the result section now includes the Calibration Intercept (alpha) and Calibration Slope (beta) with their 95% CIs for all four scores based on the complete case analysis (N=1,074).
- The Discussion has been revised to argue that the alpha approx. 0 and beta approx. 1 findings indicate a robust global calibration, thus supporting the clinical application of these scores.

***5. Clinical Utility and Cutoffs: The decision to derive new binary cutoffs using the Youden index may optimize sensitivity and specificity but might oversimplify ordinal risk categories originally proposed for the scores. The authors should justify dichotomizing continuous/ordinal scores when original tools defined multiple risk strata for triage. Also, discuss how these new cutoffs would perform in alternative settings (e.g., outpatient triage) or under varying resource constraints.***

**Response:**

We appreciate this observation, as it addresses the crucial transition from statistical findings to practical clinical utility.

1. We recognize that the original scores define multiple risk strata, but our primary objective was to perform a direct (head-to-head) and standardized comparison of the predictive capacity of the four tools for the primary binary outcome (mortality). The use of the Youden index allowed us to establish an optimal and uniform cutoff point for each score, maximizing discrimination between "high risk of mortality" and "low risk of mortality" specifically within our cohort.

2. We do not propose that ordinal scores should be dichotomized, as this will be the focus or a separate study. The dichotomization was specifically performed in order to make objective comparisons among scores.

Actions in the Manuscript:

- A clear justification for the decision to dichotomize the scores will be included in the Methods or Discussion section.

***6. Confounding and Unmeasured Variables: Mortality risk is influenced by many factors beyond those captured by scoring systems (e.g., time from symptom onset to admission, treatment availability, socioeconomic status, viral variants). Because the dataset originates from an earlier pandemic wave, factors like corticosteroid use or antiviral therapy may differ. The authors might discuss whether treatments or changes in standard of care during the study period confounded associations.***

**Response:**

We appreciate this observation, which permitted us to contextualize the performance of the scores within the dynamic and rapidly evolving environment of the pandemic.

1. We recognize that mortality is a complex outcome influenced by a multiplicity of factors that were neither included in our models nor able to be systematically captured in a retrospective study. These include fundamental variables such as socioeconomic status, the time elapsed from symptom onset to admission, and the potential influence of circulating viral variants during the study period.

2. While the objective of our work is not to formally assess the direct impact of specific treatments (such as the use of corticosteroids or antivirals), we agree that the rapid evolution of the standard of care during 2020 may have acted as a temporal confounding factor in the cohort. We mentioned in the Discussion that the consolidation of evidence regarding certain therapies during the study period may have modified the lethality of the disease in the later subset of the cohort. This variation in the risk of mortality over time, independent of the baseline score variables, is an inherent limitation of the study's retrospective nature and affects the applicability of the scores over time.

Actions in the Manuscript:

- A specific paragraph will be added to the Discussion to address unmeasured variables and the potential temporal confounding effect resulting from changes in the standard of care, without requiring a formal evaluation of the treatments.
- We will reinforce the conclusion that, due to the epidemiological and therapeutic dynamics of COVID-19, the predictive validity of any score is dynamic, not static. Therefore, our main recommendation is that periodic validation and recalibration of the scores are required for them to maintain their clinical utility over time.

***7. Sample Size Calculation and Power: The sample size calculation (582 patients) assumes 10% differences in sensitivity and specificity, but the reasoning could be better clarified. Since final included cases exceeded this threshold (1,074), power to detect smaller differences is likely adequate. However, specifying how missing data patterns affect effective sample size would help.***

**Response:**

Regarding your request for clarification regarding the sample size calculation.

1. We will specify in the Methods section that the 10% difference in sensitivity and specificity assumed for the sample size calculation was chosen because it was considered by the authors as a reasonable threshold for a clinically relevant difference that would justify the preference of one score over another in a setting of triage and risk stratification and which can be feasible. As pointed out, a post hoc analysis can show that the study had enough power to detect smaller differences.

2. We acknowledge that, while the complete-case sample for the comparison analysis was 1,074, the exclusion of a significant proportion of the original cohort reduced the total effective sample size and could introduce selection bias as there may be relevant differences among patients with and without complete data (as discussed in observation 1). The study's power was based on the complete-case analysis (n = 1,074). Although this subset provides the necessary power to compare the scores against each other, the generalization of the results is limited to this subgroup of patients with complete data, which is a limitation already addressed.

Actions in the Manuscript:

- A clear justification for the use of the 10% difference was included in the "Methods: Sample Size" section.
- It will be reinforced in the Discussion how the limitation of the analysis to complete cases affects the extrapolation of the results.

***8. Language and Clarity: While the manuscript is generally well written, there are minor grammatical errors and awkward phrases. Examples include “The outcome primary was 30‑day in‑hospital mortality” (should be “The primary outcome was 30‑day...”), and “determine” is misspelled as “determinate”. The authors should proofread to improve readability. A professional language edit is recommended before publication.***

**Response:**

We thank the reviewer for pointing out these areas that require refinement. Clarity and precision of language are essential in scientific communication.

1. Specific Corrections: We will diligently correct all identified typographical errors, grammatical mistakes, and awkward phrasing. We will ensure, for example, that phrases such as "The outcome primary was..." are corrected to the standard form "The primary outcome was..." and that the word "determinate" is corrected to "determine" where appropriate.

Actions in the Manuscript:

- A complete grammatical and stylistic review of the manuscript will be implemented.

***9. Data Availability: The supporting information or link to a repository is not provided in the PDF. For transparency, deposit the dataset and statistical code in a public repository (e.g., Dryad, OSF) and include a DOI. If ethical or legal restrictions apply (due to patient privacy), provide contact details for an institutional data access committee.***

**Response:**

We appreciate this important reminder. We confirm our full commitment to PLOS ONE’s open science and data availability policies, which require making the underlying data and code publicly available.

- We confirm that, upon potential acceptance of the manuscript, the anonymized dataset used for the analysis and the statistical code will be deposited in a public, open-access repository (e.g., Dryad or OSF) or made available upon request, according to the journal’s requirements.
