# Supplementary material for: Comparison of the performance of four clinical prediction rules for mortality in patients with COVID-19
Source: PLoS One. 2026 May 14;21(5):e0348683. doi: 10.1371/journal.pone.0348683 (PMC13175323; doi:10.1371/journal.pone.0348683)
Supplement: Attachment — Submitted filename: Response_to_Reviewers_auresp_2.doc [file pone.0348683.s007.doc]

**A rebuttal letter**

Dear Frederick K. Wangai, Academic Editor, PLOS ONE

Thank you for the opportunity to submit this minor revision. We appreciate your positive feedback regarding the strengthened data quality and calibration reporting. We have addressed each of the essential and editorial requirements point-by-point as follows:

**1. Essential revisions**

- **Missing data and selection bias (transparency):**
  - **Response:** We have explicitly stated in the **Results and Methods** that out of a source cohort of 1,963 patients, 1,074 were included in the head-to-head analysis.
  - **Action:** To address potential selection bias, we have added S2 Table, which compares the baseline characteristics of included (n=1,074) vs. excluded (n=889) patients.
  - **Clarification on Bias:** The analysis in S2 Table revealed some statistically significant differences: the included cohort was slightly older (58.2 vs 56.4 years, p=0.011) and a higher prevalence of diabetes (27.9% vs 22.7%, p=0.008). We have acknowledged these differences in the **Limitations section,** noting that our analytic sample represents a slightly higher-risk group. However, we emphasize that since all four scores were evaluated on the exact same n=1,074 patients, the relative performance and ranking of the scores (head-to-head comparison) remain internally valid and robust for this population.
  - **Action:** We provided a missingness summary in S1 Table, detailing the proportion missing for key predictors (e.g., CRP for ISARIC-4C score and LDH for CALL score) across the scores, which justifies the final analytic sample size and underscores the feasibility of simpler tools like q-CSI.
- **Cohort definitions and “head-to-head” framing:**
  - **Response:** We have clarified the distinction between the "maximal available subcohorts" (used for feasibility) and the "complete-case cohort" (used for head-to-head comparison).
  - **Action:** Tables and Results text have been relabeled to ensure clarity. We now clearly distinguish when referring to the total available data for a single score (e.g., n=1,844 for q-CSI) versus the core comparative cohort (n=1,074) used for AUROC and calibration metrics.
- **Correct interpretation of sensitivity/specificity:**
  - **Response:** We have explicitly stated in the Methods section and in the footnotes of the results text that "30-day mortality" is treated as the positive outcome.
  - **Action:** The **Discussion** (Interpretation of cutoffs) has been revised to ensure sensitivity is correctly interpreted as the ability to identify patients who will die (screening/Rule-out), and specificity as the ability to identify those who will survive (confirmatory/Rule-in).
- **Endorsement claim:**
  - **Response:** We have removed the claim that any score is "recommended by the WHO" to avoid non-endorsement issues.
  - **Action:** In the **Abstract** and **Discussion**, we rephrased this to: *"a widely validated and internationally recognized reference standard often utilized in global clinical guidelines."*

**2. Editorial/production requirements**

- **Drafting artifacts and typos:**
  - **Action:** A complete manual review of the manuscript was performed. We corrected typographical errors such as “wich” to “which” and ensured all in-text citation numbering is sequential and correctly formed. The revised file is now a clean, publication-ready version.

**3. Data availability statement**

- **Consistency:**
  - **Action:** We have updated the Data Availability Statement. S1 File now contains the complete dataset of 1,963 patients, including those with missing values, to allow for full reproducibility of our flow-chart and missingness analysis.
